# Lower-Limb Exoskeletons for Gait Training in Parkinson’s Disease: The State of the Art and Future Perspectives

**DOI:** 10.3390/healthcare12161636

**Published:** 2024-08-16

**Authors:** Matteo Fortunati, Massimiliano Febbi, Massimo Negro, Federico Gennaro, Giuseppe D’Antona, Oscar Crisafulli

**Affiliations:** 1 Department of Industrial Engineering, University of Tor Vergata, 00133 Rome, Italy; 2 CRIAMS-Sport Medicine Centre Voghera, University of Pavia, 27058 Voghera, Italy; 3 Laboratory for Rehabilitation, Medicine and Sport (LARM), 00133 Rome, Italy; 4 Department of Biomedical Sciences, University of Padua, 35131 Padua, Italy; 5 Department of Public Health, Experimental and Forensic Medicine, University of Pavia, 27100 Pavia, Italy

**Keywords:** low-profile lower-limb exoskeleton, gait training, Parkinson’s disease, disease severity, gait function, robot-assisted gait training, dual-task gait

## Abstract

Gait dysfunction (GD) is a common impairment of Parkinson’s disease (PD), which negatively impacts patients’ quality of life. Among the most recent rehabilitation technologies, a lower-limb powered exoskeleton (LLEXO) arises as a useful instrument for gait training in several neurological conditions, including PD. However, some questions relating to methods of use, achievable results, and usefulness compared to traditional rehabilitation methodologies still require clear answers. Therefore, in this review, we aim to summarise and analyse all the studies that have applied an LLEXO to train gait in PD patients. Literature research on PubMed and Scopus retrieved five articles, comprising 46 PD participants stable on medications (age: 71.7 ± 3.7 years, 24 males, Hoehn and Yahr: 2.1 ± 0.6). Compared to traditional rehabilitation, low-profile lower-limb exoskeleton (lp-LLEXO) training brought major improvements towards walking capacity and gait speed, while there are no clear major benefits regarding the dual-task gait cost index and freezing of gait symptoms. Importantly, the results suggest that lp-LLEXO training is more beneficial for patients with an intermediate-to-severe level of disease severity (Hoehn and Yahr > 2.5). This review could provide a novel framework for implementing LLEXO in clinical practise, highlighting its benefits and limitations towards gait training.

## 1. Introduction

Parkinson’s disease (PD) is a degenerative neurological disorder mainly related to the elderly (160 cases per 100,000 in ≥65-year-old population) [1,2]. Peculiarly, PD patients show gait dysfunctions (GDs) [3], which comprise, among others, a reduction in stride length, gait speed, range of movement, and interlimb asymmetry [3,4,5], mainly due to bradykinesia [6] and postural instability [7,8]. Since GD may lead to falls [3,9], PD patients restrict daily activities [10] with a consequent detrimental effect on quality of life [11], and this contributes to depressive symptoms [12], morbidity, and mortality [13]. Since walking ability is inversely associated with cardiovascular risk factors and all-cause mortality [14], and a reduction in gait function (GF) leads to an increased level of disability [15] and dependency [16], several training methodologies were studied to improve GF in PD patients [17,18,19,20,21,22]. In recent years, the development of robotic technology has impacted the world of rehabilitation and led to the development of robot-assisted gait training (RAGT) devices [17,23].

Notably, RAGT allows for partial or complete body weight support [24]. The robotic technology allows daily exercise sessions of walking practise to facilitate functional recovery and to reduce the physical burden of physical therapists [25]. There are several systematic reviews and meta-analyses on PD patients that report the usefulness of an RAGT intervention on several aspects of GFs [26,27,28,29,30], and one of them indicates superior adaptations compared to groups undergoing lower extremity rehabilitation or treadmill training in several indicators of GF in PD patients [26]. For example, from a study, the group undergoing RAGT intervention had greater enhancements as opposed to physiotherapy on walking capacity and gait speed [31], in line with a work in which such improvements were retained after a three-month wash-out period [32]. However, RAGT is not immune to intrinsic drawbacks; indeed, this technology is not portable (thus, it can only be used in a clinical setting) and has high costs [24]. To overcome these limitations, which reduce the applicability and diffusion of such methodology, an overground lower-limb wearable powered exoskeleton (LLEXO) has been implemented [33,34,35].

The LLEXO is a powered robotic apparel that moves with the joints of a human’s leg to obtain a high volume of lower-limb movements to recover or train GF [24,36]. It can replace completely or partially the work of the user, offer structural support, develop resistance to movement, or correct the trajectory [36,37,38]. The benefits of LLEXO over RAGT are that the former can be used for supervised gait training in a real environment, like at home [39,40], as well as being cheaper [41]. With such a device, patients can address the space, and, possibly, overcome obstacles encountered on the way [36]. However, despite being portable, the LLEXO can also have a considerable weight; for example, an LLEXO for motor complete tetraplegia can reach a mass of 23 kg. Interestingly, a subcategory called a low-profile lower-limb exoskeleton (lp-LLEXO), with minimal size and weight (approximately 5 kg or less) [38], has been used for gait training in populations that have the ability to walk independently [42]. Recently, LLEXO has been implemented for gait training within several pathologies like spinal cord injury (SCI) [35], acquired brain injury, and stroke [33,34]. In stroke survivors, it is reported that LLEXO is superior in the rehabilitation of GF compared to conventional training [34], while in SCI, it has superior effectiveness compared to mechanical gait orthosis [43]. However, in the clinical populations, due to the recent implementation of the overground devices, there is limited evidence of its effectiveness because of low-quality methodological studies arising from small sample sizes [44,45,46,47]. Also in PD patients, the LLEXO was introduced only recently to train GF and several questions regarding its area of application and effective usefulness still need clear answers. Some points to investigate are whether training with LLEXO could result in an improvement in walking capacity, gait kinematic variables, or spatiotemporal parameters, and on specific motor symptoms of PD patients. Moreover, up to the present date, it is unknown if LLEXO shows dissimilar effectiveness considering different stages of disease. Therefore, the aim of this narrative review is to summarise and analyse the available data regarding the use of LLEXO in PD patients in order to report emerging trends, strengths, and weaknesses; indicate gaps in knowledge; and provide new insights for future research.

## 2. Materials and Methods

### 2.1. Literature Search

This review was conducted in PUBMED and SCOPUS from April to May 2024. The research is based on work published in the past 10 years (i.e., from 2014), with the aim of detecting the new trend in robotic neurorehabilitation technologies. The search focused on works related to the actuation of a gait training intervention with an LLEXO in PD; therefore, the keywords used in the search comprised a combination of the following keywords: (“lower-limb exoskeleton”, OR “gait training”, OR “gait function”) AND “Parkinson’s disease”. Only studies regarding LLEXO assisting hip joints were considered. Five original studies, comprising four unique trials, were identified, and a brief overview is presented in Table 1.

### 2.2. Studies’ Characteristics

To date and to the best of our knowledge, five articles about the use of lp-LLEXO assisting hip joints in PD patients are available, [39,40,48,49,50]. Two studies refer to the same intervention [39,49]; thus, we will cite only Gryfe et al. (2022), instead of both, as the latter contains the comprehensive outcomes of interest. Therefore, a total of three randomised controlled trials (RCTs) and one single-group pre–post intervention were identified (Table 1). These four unique interventions have a sample size per study of 11.5 ± 6.6 subjects (mean ± SD), with a total of 46 PD participants. The mean PD stage of the disease, measured by the Hoehn and Yahr scale (H&Y), was 2.1 ± 0.6. Females represent 48% (*n* = 22) of the sample, while the males represent 52% (*n* = 24). The mean age of PD participants was 71.7 ± 3.7 years, as calculated by averaging the mean age of the lp-LLEXO groups from each study. Three studies were specifically focused on GF [40,48,50], while one primarily evaluated cognitive performances and GF was the secondary endpoint [39].

The types of lp-LLEXO treatment tested in the literature were a multi-component protocol of strength, aerobics, functional mobility, and balance [39]; a step training followed by overground walking [40]; forward walking followed by multidirectional stepping, turning, balance, and motor control tasks [48]; and a walking protocol [50]. Among RCTs, one study compared lp-LLEXO intervention with a group undergoing the same multi-component protocol without the robotic device (no lp-LLEXO) and a wait-list control group (CG), i.e., without exercise intervention [39]; Kawashima et al. (2022) matched lp-LLEXO training to a similar step and overground walking exercise (OWP), while the remaining study compared the lp-LLEXO versus a group of usual care, daily activities, and ongoing exercise regimens (UCG) [48]. The single-group pre–post study performed a within-group analysis [50]. In addition, two works evaluated the immediate changes in GF by comparing the condition of wearing versus not wearing the lp-LLEXO [40,48].

The total number of intervention sessions for each trial was not so different among the studies (range: 10–16 sessions). The duration of the intervention ranged between 4 and 12 weeks, while training frequency, precisely available in three studies, was between two [39,48] and three [50] sessions/week. One study, while not indicating a precise frequency, stated that 10 sessions spanned over 12 weeks of intervention [40]. The training time per session ranged between 20 [50] and 60 min [39,40,48]. All these studies used an lp-LLEXO during outdoor (i.e., home garden) or indoor walking training. Assessments were evaluated at baseline and at the end of the protocol in all the studies; only one work made follow-up tests after a wash-out period of 4 weeks [50]. All included investigations indicated that participants were on stable PD medication for at least 4 weeks prior to the intervention.

**Table 1:** Summary of the studies’ characteristics.

Authors	Study Type	*n*°, Participants (H&Y)	Intervention (*n*°, Participants)	Exoskeleton Type and Manufacturer (Weight)	Frequency, Protocol Duration, Session Time, Location	Main Variable Outcome
Gryfe et al., 2022, McGibbon et al., 2024 [39,49]	RCT	*n* = 13 (I = 4; II = 4; III = 5)	lp-LLEXO vs. no lp-LLEXO (*n* = 14) vs. wait-list CG (*n* = 13)	lp-LLEXO, Keeogo™	2x week, 8 weeks, 60 min, outdoor	6MWT, 10MWT, gait kinematics and spatiotemporal parameters, dual-task gait cost index, FOG-Q
Kawashima et al., 2022 [40]	RCT	*n* = 5 (II = 3; III = 2)	lp-LLEXO vs. OWP (*n* = 7)	lp-LLEXO, Honda Walking Assist Device^®^ (HWA^®^) (2.7 kg)	10 sessions within 3 months, 30 min, outdoor	3MWT, 10MWT, gait kinematics and spatiotemporal parameters, FOG-Q
Kegelmeyer et al., 2024 [48]	RCT	*n* = 20 (I = 1; II = 18; III = 4) *	lp-LLEXO vs. UCG (*n* = 20)	lp-LLEXO, Honda Walking Assist Device^®^ (HWA^®^)	2x week, 8 weeks, 45–60 min, outdoor	6MWT, gait kinematics and spatiotemporal parameters, FOG-Q
Otlet, 2023 [50]	Single group	*n* = 8 (I = 1; II = 5; 2 = 2.5)	lp-LLEXO	lp-LLEXO, Active Pelvis Orthosis (APO) (6.5 kg)	3x week, 4 weeks, 20–45 min, indoor	Gait kinematics and spatiotemporal parameters

6MWT = 6 min walk test; 3MWT = 3 min walk test; 10MWT = 10 m walk test; LLEXO = lower-limb exoskeleton gait training intervention; CG = control group; FOG-Q = freezing of gait questionnaire; H&Y = Hoehm and Yahr PD disease stage; lp-LLEXO = low-profile exoskeleton; no lp-LLEXO = same exact protocol as lp-LLEXO, but without the robotic device; OWP = similar step and overground walking training group; UCG = usual care control group. * There were 3 dropouts during this study.

### 2.3. Technical Information of lp-LLEXO

The lp-LLEXO implemented in the described studies was the Keeogo™ [39], the Honda Walking Assist Device^®^ (HWA^®^) [40,48], and the Active Pelvis Orthosis (APO) [50].

The Honda Walking Assist Device^®^ (HWA^®^) (Honda Motor Corporation, Tokyo, Japan) is an lp-LLEXO that uses the Stride Management Assist^®^ system. It extends from the hip joint to just above the knee. Precisely, it is composed of two motor and angle sensors nearly located at the hip joint, a hip frame, and two thigh frames; thus, it assists in the forward and backward extension of the leg. The HWA^®^ provides a maximum of 4 Newton·metres (N·m) of torque. It has a total mass of 2.7 kg. This device has already demonstrated clinical usefulness in stroke patients [51,52].

The Keeogo™ consists of a pelvis belt, two motors positioned laterally, and several shank and thigh cuffs to secure the device to the limb and lower trunk. It spans from the hip to just above the ankle; thus, it assists the leg and knee of the user during walking, but it does not initiate the movement; therefore, it must be worn only by a population with a certain degree of lower-limb function. The hip is allowed to rotate freely when wearing the Keeogo™. It has been proven to have beneficial effects in restoring GFs in a cohort of different neurological patients [53], in MS [42], and in people with osteoarthritis [54].

The Active Pelvis Orthosis (APO, IUVO, Pisa, Italy) is composed of a horizontal C-shaped frame, surrounding the user’s hips, and the back of the pelvis interfaces with the user’s trunk by means of three orthotic shells (two lateral and one rear) and carries two actuation units mounted on the lateral arms [55]. The APO assists in the flexion and extension of the hip; thus, it helps control the leg. The device does not assist with weight support or balance [56]; therefore, it must be used by individuals with a certain degree of lower-limb and trunk function. The maximum torque is 35 N·m, and its mass is 6.5 kg [56]. The usefulness of APO has already been reported in a cohort of patients with acquired brain injury [56], and in the elderly [57].

### 2.4. Outcome Measures

Two studies evaluated GF through the 6 min walk test (6MWT) [39,48], while one monitored the shorter version of three minutes (3MWT) [40]. The 6MWT is commonly used in clinical populations [58], is recommended by the Movement Disorder Society (MDS) for PD patients, and is proposed as a valid tool to assess the ability to walk outdoors [59].

All studies evaluated GF through gait kinematics and spatiotemporal parameters. Specifically, gait speed [39,40,48,50], stride length [48,50] and its coefficient of variation (CV) [48], step length [40], stride duration [50], cadence [40], swing time and its CV [48], double support time and its CV [48], flexion/extension range of motion (ROM) at the thighs with symmetry of mobility among them [40], range of the scissor angle on both thighs with symmetry of the scissor angle between them [40], and ROM at the hip level [50] were measured. Overall, gait kinematics and spatiotemporal parameters are important factors in evaluating the consequences and the progression of neurological disorders [60,61,62,63,64]; moreover, in PD, they are associated with an increased risk of falls [65]. The evaluation of gait speed, measured using the 10 m walk test (10MWT), is a recommended evaluation in PD patients by MDS [59]; indeed, it predicts falls [66,67]. Stride length, a parameter composed of two consecutive step lengths, is strongly associated with cadence [68] and, again, it is associated with the risk of falls [69]. Stride length CV is increased in PD patients compared to age-matched healthy controls [70]. Swing time and double support time in male PD patients increase with the progression of the disease [71]. Lower-limb ROM is reduced in PD patients [72] and it is helpful in detecting the motor asymmetry (i.e., unilateral progression) of the disease [73,74].

Only one study assessed the dual-task (DT) gait cost index through a motor assignment [39] during 10MWT, with gait speed timed within the inner 6 m following the guidelines of the Canadian Consortium on Neurodegeneration and Aging [75]. In fact, DT, which involves an additional cognitive load/tasks (such as arithmetic, language, memory) on top of the gait locomotor task during walking [76,77], can represent a further concern for PD patients, due to its influence on the performance of several gait-related parameters (e.g., speed, stride length, cadence) or motor tasks during walking.

Finally, three studies assessed the freezing of gait (FOG) symptom [39,40,48] through its short, reliable, and valid questionnaire (FOG-Q) [78] that is recommended by the MDS [59]. The FOG consists of an unpredicted and brief episode, usually lasting <10 s, in which a PD patient moves forward with small steps, trembles in place, or presents total akinesia [79,80,81], and it is positively associated with the risk of falls [9].

## 3. Results

### 3.1. Effectiveness of lp-LLEXO Training on Outcome Measures

#### 3.1.1. Effectiveness of lp-LLEXO Training on the Six-Minute Walk Test

Immediate effect of wearing lp-LLEXO: Donning an lp-LLEXO reduced 6MWT, although not significantly [48].

**Figure 1:**
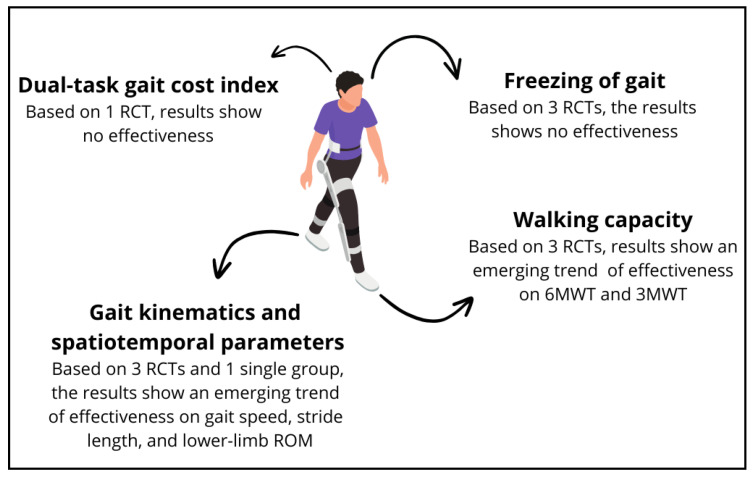
Pre-to-post within lp-LLEXO training effectiveness on outcome variables. Notably, the presented figure represents only the SMA device.

Pre-to-post within lp-LLEXO training: One study showed a significant improvement in 6MWT [39], while the increase reported by Kegelmeyer et al. (2024) [48] did not reach statistical significance. The only work that evaluated 3MWT found a significant gain in distance [40] (Figure 1).

Between lp-LLEXO training and control groups: The 6MWT was not statistically different among lp-LLEXO and no lp-LLEXO, while it reached a statistically significant improvement versus wait-list CG; however, both control groups had a marginal decrease in metres walked, while the lp-LLEXO group had an amelioration [39]. The remaining studies found no significant differences in 6MWT between lp-LLEXO and UCG [48] and in 3MWT among lp-LLEXO and OWP; however, in the first control group, there was a similar increase compared to LLEXO [48], while, in the latter, the performance slightly decreased [40] (Table 2).

#### 3.1.2. Effectiveness of lp-LLEXO Training on Gait Kinematics and Spatiotemporal Parameters

Immediate effect of wearing lp-LLEXO: In one study, wearing an lp-LLEXO significantly improved gait speed, step length, flexion/extension ROM of both thighs, and range of the scissor angle on both thighs, while it was not able to increase with a cut-off *p*-value cadence, nor to modify the symmetry of mobility or symmetry of the scissor angle among thighs [40]. On the contrary, in the second work, donning an lp-LLEXO reduced gait speed, stride length, swing time, and double support time CV, while it did not modify stride length CV and swing time CV, and increased double support time. However, not one of these changes was of statistical significance [48].

Pre-to-post within lp-LLEXO training: All studies showed an improvement in gait speed, with only one reporting a significant *p*-value [50]. Two works found an increase in stride length [48,50], but only one reached a statistically significant result [50]. Furthermore, step length [40], stride length, and swing time were improved [48]; swing time CV and double support time and its CV were reduced [48]; and there was no change in stride length CV [48]. However, these results were all non-significant [40,48]. Finally, there was a statistically significant improvement in stride duration and ROM at the hip level [50]. Moreover, gains in gait speed, stride length, stride duration, and hip ROM were maintained after a wash-out period of weeks [50]. The increase in gait speed, stride length and duration, and hip ROM can be maintained after a 4-week period [50] (Figure 1).

Between lp-LLEXO training and control groups: Gait speed was not significantly different among lp-LLEXO and no lp-LLEXO, wait-list CG [39], and UCG [48]; however, the LLEXO group shows a superior conditioning versus all control groups. In addition, the LLEXO group was not significantly different from OWP [40], but gait speed decreased in the latter [40]. Stride length and its CV, swing time and its CV, and double support time and its CV were not statistically different among lp-LLEXO and UCG [48]. Step length did not show any statistical change between lp-LLEXO and OWP [40] (Table 2).

#### 3.1.3. Effectiveness of lp-LLEXO Training on Dual-Task Gait Cost Index

Pre-to-post within lp-LLEXO training: The DT gait cost index showed an increase in its score, reflecting a worsening trend in the locomotor task; however, it did not reach a level of statistical significance [39] (Figure 1).

Between lp-LLEXO training and control groups: The DT gait cost index decreased only in the wait-list CG, while it increased in both lp-LLEXO and no-lp-LLEXO groups. The between-group comparison showed only a statistically significant difference among lp-LLEXO and wait-list CG [39] (Table 2).

#### 3.1.4. Effectiveness of lp-LLEXO Training on Freezing of Gait

Pre-to-post within lp-LLEXO training: The FOG-Q, even if slightly decreased (i.e., the symptom improved), was not statistically significant in two studies [39,40], while it trended to a slight increase (i.e., the symptom worsening) in the remaining studies [48]. However, such changes were not statistically significant (Figure 1).

Between lp-LLEXO training and control groups: The FOG-Q was not statistically different among lp-LLEXO and no lp-LLEXO or wait-list CG; however, in the latter, its score worsened [39]. The remaining studies found no significant changes among lp-LLEXO and UCG [48] or versus OWP, but in the control group, it highly exacerbates the symptom reflected by an increase in the questionnaire score [40] (Table 2).

## 4. Discussion

### 4.1. Summary of Evidence

To the best of our knowledge, this is the first review summarising the effectiveness of lp-LLEXO on GF in patients with PD.

Taken together, the characteristics of the lp-LLEXO intervention (total and weekly duration, frequency, modality) are somehow homogeneous within the literature screened as well as the type of intervention among the studies [39,40,48,50]; only Gryfe et al. implemented some strength, balance, and functional mobility in addition to a walking practise. Overall, from a within-group perspective, the available data would suggest a substantial usefulness of lp-LLEXO training towards GF, as highlighted by the ameliorations reported within a short-term protocol (≤12 weeks or 10–16 sessions) in the 6MWT, 3MWT, and several gait kinematics and spatiotemporal parameters among the different cohorts analysed. Instead, no significant effectiveness has been reported for the DT gait cost index and FOG-Q. In addition, from a between-group comparison, it seems that lp-LLEXO intervention shows similar adaptations to control groups in several gait kinematics and spatiotemporal parameters (i.e., stride and step length), but superior effectiveness in the 3- and the 6-MWT, and in gait speed. Furthermore, when it comes to the FOG-Q and DT gait cost index, while the first showed no statistically significant difference, the latter statistically worsened compared to wait-list CG.

The lp-LLEXO training intervention for gait training in PD patients reported in this review is cohesive with new trends and innovations in neurorehabilitation technologies [82,83,84]. Specifically, there are several examples of proof of its effectiveness in stroke rehabilitation [84], SCI patients [85], and MS [86]. Indeed, there is a growth in the implementation of LLEXO in clinical populations to restore GFs to allow activities of daily living, which translates to improved freedom, quality of life, and health [87].

### 4.2. Effectiveness of lp-LLEXO Training versus Outcome Measures

#### 4.2.1. Effectiveness of lp-LLEXO Training on the Six-Minute Walk Test

Considering the outcomes, lp-LLEXO intervention shows a favourable trend in improving the distance walked in tasks ≤6 min [39,40,48] and, based on our limited evidence, could be more beneficial than traditional rehabilitation methods. A reason for the greater improvement in 6MWT evaluated by Gryfe et al. is that lp-LLEXO was able to progressively increase its training intensity throughout the intervention, while no lp-LLEXO plateaued after 4 weeks (mid-phase) [49]. Moreover, Kegelmeyer et al. suggested that the significant result of Kawashima et al. could also be attributed to a greater proportion of PD patients with a severe stage of the disease who entered the lp-LLEXO group compared to OWP, who might have benefited more from the assistance of the robotic device. Kawashima et al. reported that the HWA^®^ device might automatically reinforce the function of spinal central pattern generators with a small effort of the participants and could facilitate sensory feedback via proprioceptive and skin afferents [87,88], thus resulting in an increase in walking capacity. These results are in line with several studies on different pathologies, like SCI [47], and multiple sclerosis (MS) [89], and with other robotic devices among PD patients (i.e., RAGT) as well [26]. That is, the expected increase in walking distance through lp-LLEXO intervention in PD patients can exceed the minimal clinically important difference (MCID) in adults with pathology [90], which may translate into a reduced mortality risk [91,92], and an increase in independence [16]. Finally, the lack of a beneficial effect on 6MWT among the immediate condition of wearing/not wearing the lp-LLEXO may be due to the insufficient time to become familiarised with the robotic device [48]. This would be cohesive with the data reported for SCI patients, in which the rate of learning for the utilisation of the LLEXO device varies between participants [93].

#### 4.2.2. Effectiveness of lp-LLEXO Training on Gait Kinematics and Spatiotemporal Parameters

Gait speed increased in all the studies even though it was not statistically significant in most of them [39,40,48]. Only one study [40] showed that the improvement was above the MCID [94]. Importantly, Gryfe et al. suggested that their participants were already well conditioned, and this may have been a determent in obtaining statistically significant results; indeed, the baseline gait speed of said group was higher than the normative reference value in older healthy adults [95]. However, the absence of statistical significance could also be due to the fact that Gryfe et al. indirectly calculated gait speed by measuring the time to walk at a fixed distance with a stopwatch, a procedure that may have a higher rate of error compared to other methodologies as previously suggested [96]. Moreover, the outcome may suggest that LLEXO shows a superior trend in effectiveness versus traditional gait training rehabilitation methods; indeed, two control groups showed a less absolute increase in gait speed than LLEXO [39,48], and one reported a slight decrease [40]. Again, lp-LLEXO training might have improved gait speed through the reinforcement of the function of spinal central pattern generators with a small effort of the participants and could facilitate sensory feedback via proprioceptive and skin afferents [87,88]. Furthermore, (not statistically significant) improvements in self-selected gait speed following an LLEXO intervention have been observed in SCI [47] and MS as well [97].

Stride length significantly improved in one study [50], while Kegelmeyer et al. reported a non-significant increase. However, in the latter investigation, lp-LLEXO training trended to an increase in stride length that was much more beneficial in PD patients with advanced stages of the disease. That is, PD participants at an early stage of the disease might have minimal room for improvement compared to PD patients at advanced stages. In fact, their participant group was composed mostly of PD patients at an early stage of the disease [48].

Step length did not show any changes in one work [40]; however, the lp-LLEXO group showed longer step length at the baseline assessment than the control, which could translate into a non-significant improvement after the intervention due to the participants’ level, likely already well conditioned. In other pathologies such as SCI, the step length of the right leg increased significantly from pre-to-post intervention [46], as well as in comparing training with LLEXO as opposed to traditional orthoses [98].

In PD patients, hip ROM improved from pre-to-post intervention [50] and during an immediate condition from donning/doffing the robotic device (Kawashima et al., 2022 [40]). In SCI patients, walking with an LLEXO increases knee ROM compared to traditional orthoses [98].

To the best of our knowledge, no other studies with LLEXO evaluated stride length CV [48], stride duration [50], cadence [40], swing time and its CV [48], double support time and its CV [48], range of motion at the thighs with symmetry of mobility among them [40], range of the scissor angle on both thighs with symmetry of the scissor angle between them [40], and ROM at the hip level [50].

Taken together, mounting evidence hints towards lp-LLEXO as an intervention holding the potential to increase several gait kinematics and spatiotemporal parameters in PD patients, which, in turn, might also translate into an MCID change as well. Notably, it seems that PD patients already within an average gait speed performance and stride length would not be suitable for significant lp-LLEXO training-related benefits.

#### 4.2.3. Effectiveness of lp-LLEXO Training on Dual-Task Gait Cost Index

The DT gait cost [76,77] is due to cognitive–motor interference; indeed, patients with PD must use a greater amount of their cognitive resources to walk and, therefore, when a concomitant activity is carried out, the cortical resources’ allocation mismatch leads to a deficit in forward progression [99]. Regarding Gryfe et al., the DT gait cost index of a motor assignment in 10MWT improved only in wait-list CG, but without a statistical amelioration, while its score worsened in lp-LLEXO and no lp-LLEXO. Thus, based on the results, no effectiveness of lp-LLEXO on DT gait cost was found. Interestingly, only lp-LLEXO had a significant improvement in the Scale for Outcomes Parkinson’s Cognition post-test, while both control groups had only a marginal augment and, moreover, the MDS-UPDRS score of lp-LLEXO worsened less than either no lp-LLEXO or wait-list CG. Motor–cognitive training is beneficial in reducing the overall psychophysiological burden of DT (i.e., gait speed, cadence, and stride length) in PD patients [100], and an RAGT intervention with auditory and visual cueing seems superior in improving gait speed under a cognitive task compared to treadmill training [101]. Based on such evidence, it seems plausible that lp-LLEXO intervention might have been superior in the DT gait cost index versus no lp-LLEXO and wait-list CG. In fact, it might be speculated that a robotic device, mechanically guiding the limb through fixed movements, could influence cognitive resources used for walking. For instance, a recent study showed that a motor–cognitive training (i.e., exergame) intervention was able to facilitate the neural drive to the ankle dorsiflexor in DT gait, although in healthy older adults [102]. Thus, PD patients would have been better long-term-conditioned, and, on a theoretical basis, it may be even better than a traditional conditioning DT programme without lp-LLEXO and a wait-list CG.

#### 4.2.4. Effectiveness of lp-LLEXO Training on Freezing of Gait

The FOG symptom slightly improved in the lp-LLEXO group in two studies, even without reaching a statistical significance [39,40]; however, the FOG symptom slightly worsened in the third investigation [48], while in the control groups of all the studies, the symptom was aggravated [39,40,48]. Thus, such results, gathered together, suggest that lp-LLEXO has no effectiveness in treating FOG symptoms in PD patients. However, the observed non-significant improvement obtained from pre-to-post interventions could be attributed to the low dose of the training (e.g., 16 sessions in eight weeks [39], 10 sessions within three months [40]) or, as suggested by Gryfe et al., that their PD participants of the lp-LLEXO group already had a low impaired functional level, which translated into an increased difficulty in improving the questionnaire scores. So far, it is well known that FOG is reduced by physical therapy [103], which can explain why wait-list CG reported an increase in the questionnaire score [39]. Moreover, when RAGT intervention is considered, it results in better treating FOG compared to treadmill training [104].

Thus, it might be conceivable that an lp-LLEXO, due to its mechanical drive of the legs, which act like a cueing system [105], has the potential to improve FOG symptoms compared to traditional physical therapy. The reason is that PD patients will be helped from lp-LLEXO during the symptoms (short stepping, freezing, or trembling) by maintaining a constant and rhythmic cadence of walking, thus allowing an intensive training [104].

#### 4.2.5. Effectiveness of lp-LLEXO Training within Parkinson’s Disease Severity

Interestingly, Kegelmeyer et al. discovered that stride length improved to a greater extent in PD patients with a more severe stage of the disease (i.e., H&Y > 2.5) compared to H&Y < 2, while Gryfe et al. suggested that lp-LLEXO training might have had a statistical effect if the functional impairment level of the participants had been higher. Taking these results together, it might seem reasonable to think that an lp-LLEXO has the most benefit in training GF with an intermediate-to-severe level of disease severity (H&Y > 2.5) or PD patients with a mid-to-high level or score in GDs. For PD patients with less disease severity or higher functionality scores, active training might still hold the most potential. This speculation can be corroborated by the results observed in several other investigations where participants suffering from different pathologic conditions [44,106,107]. For instance, SCI patients were still able to walk a few metres wearing an LLEXO [44,106], although they had lost their motor and sensory functions in the lower limb.

#### 4.2.6. Lp-LLEXO Training Improvements in Quality of Life

Interestingly, several studies, in combination with GFs, have investigated the quality of life using several scales, questionnaires, or evaluations, such as the Movement Disorder Society—Unified Parkinson Disease Rating Scale (MDS-UPDRS). Indeed, regarding MDS-UPDRS, an instrument specifically designed for PD patients [108], it was assessed in two studies [39,40]. In terms of Gryfe et al., there was a worsening in the score in lp-LLEXO, no lp-LLEXO, and wait-list CG; however, the increase in points was almost the same for the active treatments, while the change was nearly double in the inactive group. The research team suggested that the low disability level of the sample recruited could have led to a non-statistical change in the MDS-UPDRS score [39]. Regarding Kawashima et al. [40], the MDS-UPDRS decreased (symptoms ameliorated) in lp-LLEXO, while they marginally worsened in OWP. These results, taken together, indicate an uncertain effectiveness of lp-LLEXO training on the specific MDS-UPDRS scale; therefore, based on this limited evidence, we may assume a trivial benefit in daily living. However, comprising other health-related scales, Gryfe et al. show that lp-LLEXO intervention, along with or due to GF improvements, largely ameliorates PD-related health, mobility, activities of daily living, emotional well-being, social support, cognition, and communication compared to no lp-LLEXO [39]. In addition, Kawashima et al. [40] also reported that both interventions (lp-LLEXO training and OWP) improved PD-related health status; however, in the latter, the improvement was minor. Therefore, if we consider the outcome in MDS-UPDRS added to other health-related scales, we can suggest a slight trend in the effectiveness of lp-LLEXO training compared to control groups. Such benefits are in line with those deriving from traditional rehabilitation methodologies [109,110,111,112].

## 5. Conclusions

Overall, based on the literature screened but acknowledging that more studies are needed to disentangle the role of lp-LLEXO in PD patients’ training, there is an emerging trend of a beneficial effect in PD patients following lp-LLEXO training, involving 3- and 6-min walking tests, gait kinematics, and spatiotemporal parameters. Moreover, results from the screened literature hint towards a potential more effective role of lp-LLEXO in training PD patients with intermediate–severe GD or H&Y mid–severe stages (≥2.5).

## 6. Limitations and Future Directions

The following research has several limitations, starting from the paucity of results due to a low number of studies and participants per study. Up to the present date, only lp-LLEXO has been implemented in early-to-severe PD patients; thus, it would be useful to evaluate LLEXO training effects to GF in different stages of H&Y. That is, existing knowledge allows for the speculation that an LLEXO-based training may bring fewer results in PD patients with an early/moderate stage of the disease compared to a more advanced stage of the pathology. Additionally, another major limitation is the heterogeneity of lp-LLEXO used in the studies; indeed, only two works implemented the same device (HWA^®^) [40,48]. Thus, also given the difference in the type, design, and contribution to movements of the orthosis, it leads to different stimuli in PD participants, leading to different adaptations. Therefore, future work may compare GF outcomes based on different types, designs, and properties of LLEXOs to detect specific benefits arising from their unique intrinsic characteristics. Interestingly, among the screened studies, there is no investigation evaluating the impact of LLEXO on functional mobility measures such as the “Timed Up and Go” test, which is a common, valid, easy to administer, and reliable endpoint in clinical [113] and PD patients [59]. For instance, this evaluation would help assess postural instability and GD [114]. However, future studies are needed to shed light on the effectiveness of LLEXO on FOG, a major concern in PD patients [80], which is also associated with a higher risk of falling [9]. Moreover, there is uncertainty about the benefits of LLEXO training on the DT gait cost index; therefore, it would be necessary to investigate if this intervention strategy can improve DT as seen with RAGT [101]. Considering that a pilot study has shown that in PD patients, the type of conditioning during DT can exert different adaptations [77], an investigation evaluating in which area DT with LLEXO training can have the most potential would be crucial. Also, because DT gait cost can be assessed through several variables [100], an investigation focusing on LLEXO training on more than one outcome variable is requested. In addition, no studies evaluated the modification of peak oxygen consumption and pulmonary functional tests among LLEXO training in PD. This has already been performed within SCI, and it was found that LLEXO intervention has a positive association with several spirometry measures, such as forced vital capacity [106]. These outcomes would be useful in evaluating the cardiovascular effectiveness of the intervention. In addition, another limitation is that there are no studies that compare the benefits in GF when using RAGT compared to LLEXO as was performed in SCI [47]; such an investigation would be helpful to address further studies evaluating the robotic-device-dependent domain gains. Moreover, since cognitive impairment is a peculiar trait of PD [115], such patients may find it difficult to wear and use the LLEXO or lp-LLEXO by themselves, thus requesting a qualified figure nearby. The issue arises from the observation of Khan et al. (2019), which reported a slow rate of learning for basic movements in a cohort of clinical patients without cognitive impairments [93]. Up to the present date, however, no studies have investigated the rate of learning among LLEXO and its low-profile version in PD patients; thus, it must be assessed with a proper design. Furthermore, an investigation discovering potential mechanisms involved in the beneficial effects of LLEXO training on gait function is requested. In addition, when the studies implementing LLEXO in PD patients will be sufficient, an analysis that stratifies the results by different ages (as well as time since first diagnosis), genders, and characteristics of the training intervention (e.g., 2 versus 3 times/week, short versus long intervention, etc.) will be needed. Furthermore, a work investigating the perceived benefits and health-related and quality-of-life outcomes emerging from an LLEXO intervention in PD patients is requested, as few studies have been conducted to draw conclusions. Finally, research on long-term improvements’ retention is required to compare them with respect to the traditional rehabilitation methods [116].

## Figures and Tables

**Table 2:** Within-group main variables’ outcome after the treatments.

Infographic	Test	lp-LLEXO	Active Control Group	Passive Control Group
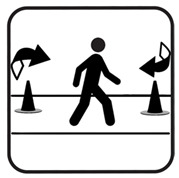	6MWT	from 374.4 ± 78.9 m to 409.3 ± 90.8 m, *p* < 0.001 [39] from 401.3 ± 136.7 m to 416.9 ± 124.3 m, *p* > 0.05 [48]	from 369.3 ± 122.0 m to 367.9 ± 120.3 m, *p* = 0.8 [39] from 403.6 ± 109.7 m to 426.9 ± 125.0 m, *p* > 0.05 [48]	from 354.6 ± 117.1 m to 350.9 ± 117.0 m, *p* = 0.7 [39]
	3MWT	from 141.4 ± 14.4 m to 154.7 ± 15.9 m, *p* = 0.02 [40]	from 142.5 ± 17.9 m to 142.1 ± 17.0 m, *p* = 0.9 [40]	
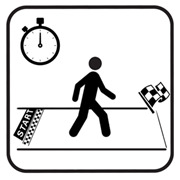	10MWT	from 1.16 ± 0.23 m/s to 1.21 ± 0.19 m/s, *p* = 0.2 [39] from 0.90 ± 0.13 m/s to 1.01 ± 0.17 m/s, *p* = 0.16 [40] from 1.19 ± 0.3 m/s to 1.25 ± 0.3 m/s, *p* > 0.05 [48] from 0.97 m/s to 1.25 m/s to 1.27 m/s, *p* ≤ 0.001 [50]	from 1.16 ± 0.23 m/s to 1.21 ± 0.19 m/s, *p* = 0.2 [39] from 0.85 ± 0.14 m/s to 0.84 ± 0.13 m/s, *p* = 0.8 [40] from 1.16 ± 0.2 m/s to 1.25 ± 0.3 m/s, *p* > 0.05 [48]	from 1.14 ± 0.32 m/s to 1.16 ± 0.35 m/s, *p* = 0.7 [39]
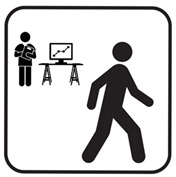	Step length	from 43.9 ± 1.7 cm to 44.5 ± 4.3 cm, *p* = 0.8 [40]	from 33.2 ± 0.0 cm to 32.3 ± 0.0 cm, *p* = 0.5 [40]	
	Stride length	from 124.4 ± 27.2 cm to 128.9 ± 25.5 cm, *p* > 0.05 [48] from 1.25 m to 1.29 m to 1.30 m, *p* ≤ 0.001 [50]	Stride length: from 124.8 ± 24.0 cm to 131.7 ± 30.8 cm, *p* > 0.05 [48]	
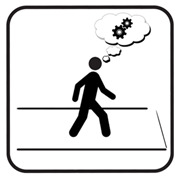	Dual-task gait cost index	from 16.0 ± 15.6% to 21.5 ± 14.8%, *p* = 0.06 [39]	from 13.6 ± 9.2% to 17.3 ± 11.4%, *p* = 0.013 [39]	
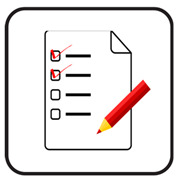	FOG-Q	from 7.6 ± 5.7 a.s. to 6.8 ± 6.5 a.s., *p* = 0.4 [39] from 12.2 ± 1.6 a.s. to 11.4 ± 1.7 a.s., *p* = 0.3 [40] from 8.5 ± 6.9 a.s. to 9.0 ± 6.4 a.s., *p* > 0.05 [48]	from 7.3 ± 6.2 a.s. to 6.8 ± 5.1 a.s., *p* = 0.5 [39] from 8.4 ± 1.2 a.s. to 10.6 ± 1.8 a.s., *p* = 0.2 [40] from 9.18 ± 4.6 a.s. to 9.85 ± 5.4 a.s., *p* > 0.05 [48]	

6MWT = 6 min walk test; 3MWT = 3 min walk test; 10MWT = 10 m walk test; a.s. = absolute score; FOG-Q = freezing of gait questionnaire; *p*-value = within group.

## Data Availability

Not applicable.
